# Development of the core of an ICF-based instrument for the assessment of work capacity and guidance in return to work of employees on sick leave: a multidisciplinary modified Delphi study

**DOI:** 10.1186/s12889-022-14653-0

**Published:** 2022-12-28

**Authors:** Astrid de Wind, Birgit H. P. M. Donker-Cools, Lyanne Jansen, Clare H. Luymes, Sylvia J. van der Burg-Vermeulen, Shirley Oomens, Johannes R. Anema, Frederieke G. Schaafsma

**Affiliations:** 1 Amsterdam UMC location University of Amsterdam, Public and Occupational Health, Amsterdam, the Netherlands; 2 Amsterdam Public Health research institute, Societal Participation and Health, Amsterdam, the Netherlands; 3 Research Center for Insurance Medicine, Amsterdam, The Netherlands; 4 HAN University of Applied Sciences, Occupation and health research group, Arnhem, the Netherlands; 5 Radboudumc, Department of Primary and Community Care, Nijmegen School of Occupational Health, Nijmegen, the Netherlands; 6 Amsterdam UMC location Vrije Universiteit Amsterdam, Public and Occupational Health, Amsterdam, the Netherlands

**Keywords:** Delphi technique, Disability, Return to work, Sick leave, Work capacity

## Abstract

**Background:**

Several occupational health disciplines are involved in return to work guidance, implying that good interdisciplinary collaboration is important. A shared conceptual framework and a common language for the assessment of work capacity and guidance in return to work is expected to be at the benefit of appropriate and sustainable employability of sick employees. The International Classification of Functioning, Disability and Health (ICF) can be considered a shared conceptual framework and is also promising in terms of a common language. The purpose of the current study is to reach multidisciplinary consensus among occupational health professionals on the content of an ICF-based instrument for the assessment of work capacity and guidance in return to work.

**Methods:**

To obtain multidisciplinary consensus we conducted a modified Delphi study among twelve occupational health experts, including four occupational physicians, four insurance physicians and four labour experts. The study included two e-mail rounds and two virtual meetings. In the consecutive rounds the experts assessed ICF items as well as a list of non-ICF-based work-related environmental factors on their relevance for the assessment of the work capacity and guidance in return to work together with their interpretability.

**Results:**

The four consecutive Delphi rounds resulted in 20 items that are minimally needed for the assessment of the work capacity and return to work possibilities of employees on sick leave. The final list included six items on personal functioning, seven items on social functioning and seven items on physical functioning.

**Conclusions:**

This set of items forms the core of an ICF-based instrument, which is expected to facilitate interdisciplinary and intradisciplinary communication because of the use of a shared conceptual framework. As such, it should be of help in the guidance in return to work of employees on sick leave and contribute to appropriate and sustainable employability.

**Supplementary Information:**

The online version contains supplementary material available at 10.1186/s12889-022-14653-0.

## Background

In occupational healthcare a paradigm shift is taking place from a biomedical perspective on employees on sick leave towards a biopsychosocial perspective entailing an interactional and holistic view of disability. An interactional view of disability acknowledges that disability is determined by the interaction between a person’s body functions and the persons’ (social) environment [1]. Beyond biomedical factors, attention is given to personal and environmental factors, that may play a role with regard to the work capacity of an employee on sick leave and the return to work possibilities. A second and related shift is taking place for the work capacity assessment of disability benefit claimants, which is shifting from a focus on disability and incapacity, towards a broader approach also entailing the assessment of remaining work capacity [1, 2]. This new approach for the assessment of work capacity fits within a larger context with labour market policies aiming at individuals experiencing health problems to remain active in work [3]. These two shifts in thinking in occupational healthcare underline the importance of return to work guidance from an integral perspective.

In the Netherlands, several occupational health disciplines, i.e. occupational physicians (OPs), labour experts (LEs) and insurance physicians (IPs), are involved in the assessment of work capacity to the benefit of return to work guidance (see Box 1), implying that good interdisciplinary collaboration is of utmost importance. In general, work capacity is understood as ‘the overall ability of an individual to perform the physical, mental and emotional tasks that are needed for the requirements of a particular job, or class of jobs’ [3]. However, the different occupational health disciplines have different roles and responsibilities [4] as well as different conceptual frameworks when it comes to the guidance of employees on sick leave [5], which can be challenging in the collaboration. Different perspectives on the work capacity may be harmful for the return to work of these employees as well as their health recovery on the long term. A shared conceptual framework and a common language for the assessment of work capacity and guidance in return to work is expected to be at the benefit of appropriate and sustainable employability of sick employees.

The International Classification of Functioning, Disability and Health (ICF) can be considered a shared conceptual framework and is also promising in terms of a common language [6]. When the ICF framework is applied to employees on sick leave, work capacity and return to work possibilities are an outcome of the interaction of an employee’s health problem with personal and environmental factors. Also, with its defined categories the ICF provides a language about health and functioning for interdisciplinary communication in the context of occupational healthcare. The potential of the ICF for occupational healthcare can be derived from the fact that it has already been integrated into several existing practice-based instruments (e.g. the Social Medical Work Capacity instrument from the Netherlands [2] and the Activity Capacity Assessment from Sweden [7]).

The Dutch Ministry of Social Affairs and Employment requested the research team to develop an instrument for the assessment of work capacity and guidance in return to work to be used during the first two years of sick leave within the Dutch occupational healthcare system, which is designed in accordance with the Gatekeeper Improvement Act [8, 9] [see Box 2]. The instrument should be based on a shared conceptual framework and a common language. As such, it should facilitate the interdisciplinary collaboration between OPs, LEs and IPs in the guidance of employees on sick leave.

All in all, the ICF seems promising to serve as base for the development of an instrument with a common language for the assessment of the work capacity and return to work possibilities of employees on sick leave. However, with 1424 categories the ICF is too extensive to apply in occupational healthcare as an assessment and guidance instrument in daily clinical practice. To date it is unknown which ICF categories are minimally needed for the assessment of the work capacity and return to work possibilities according to occupational health professionals. Therefore, the aim of the current study is to reach multidisciplinary consensus among occupational healthcare professionals on the content of such an ICF-based instrument by conducting a modified Delphi study.

Box 1. The roles of the different occupational health disciplines involved in return to work guidance and work disability assessment in the Netherlands **During the first two years of sick leave**

• The occupational physician advises on the work capacity of an individual in relation to the current job situation. He or she guides both the employee and employer in the return to work process

• The labour expert uses the information provided by the occupational physician to assess return to work options in case an employee may no longer be able to fully return to the current job situation. Both the occupational physician as well as the labour expert take into account relevant work and personal factors that may hinder return to work

**From return to work guidance to work disability assessment**

• The insurance physician at the social security agency will assess the general work capacity of the individual in comparison to a healthy person with a similar age and educational background

• Based on that assessment, the labour expert within the social security agency will then assess the remaining earning capacity of the individual and calculates if disability benefits are applicable

Thus, while the occupational physician and the labour expert during the first two years of sick leave adopt a vocational rehabilitation perspective, the insurance physician and the labour expert from the social security agency adopt a social security perspective

Box 2. Gatekeeper Improvement Act [8, 9] According to the Gatekeeper Improvement Act employers, employees and occupational physicians are obliged to follow several steps to promote soon return to work:

• The employee calls in sick during the first day of sick leave

• In case of prolonged sick leave after six weeks, an occupational physician conducts a problem analysis of the reasons for sick leave and necessary interventions and guidance for return to work

• Based on the problem analysis the employer and employee together agree on an action plan for return to work, including necessary work modifications

• In case of prolonged sick leave after one year, the employer facilitates the search for another job in another organization

• In case of prolonged sick leave after two years, the employee applies for disability benefits through the social security agency

## Methods

### Study design

We conducted a modified Delphi study to obtain multidisciplinary consensus among professionals involved in the guidance in return to work of employees on sick leave. Our modified Delphi study concerned a combination of rounds in line with Delphi principles complemented with virtual face-to-face meetings. The Delphi technique is commonly used to reach consensus in healthcare in case of inadequate or lacking published information [10]. It is characterized by collecting opinions among a group of anonymous experts taken in a series of rounds [10, 11]. The face-to-face group meetings were conducted in an online environment due to COVID-19 circumstances and social distancing measures that were in place at the time of data collection. The development of the ICF-based instrument has been presented as part of the background document of a Dutch guideline (under development) for occupational physicians, insurance physicians and labour experts [12, 13].

### Expert panel and research team

A multidisciplinary expert panel was composed by the research team and represented all relevant professional groups within the domain of occupational healthcare: four occupational physicians (OPs), four insurance physicians (IPs) and four labour experts (LEs). They were recruited through their professional associations. They were eligible when 1) being a (formerly) practicing OP, IP or LE (and as such have practice expertise in the assessment of work capacity and/or return to work guidance) and 2) willing to participate on personal title and not on behalf of their professional association. Ten of the experts were still practicing their occupation at the time of the research. The research team itself also consisted of experts within occupational healthcare, including both occupational health- and insurance physicians.

### Composition of a list of ICF categories

To apply the ICF in clinical practice, ICF core sets have been developed that are relevant for specific health conditions or within specific healthcare contexts [14]. In order to compose a list of potentially relevant ICF categories for the domain of occupational healthcare the research team searched in the scientific literature for relevant ICF core sets and ICF based practice instruments and informed within their own network of researchers and occupational health experts. The following ICF core sets and practice-based instruments were identified: core set disability evaluation [15], core set vocational rehabilitation [16] and the Social Medical Work Capacity assessment instrument [2]. Based on ICF categories derived from these core sets the research team composed a list of potentially suitable ICF categories. This list was expanded with ICF categories present in > 70% of disease specific core sets, e.g. musculoskeletal conditions [2]. In addition, all unique ICF categories from the disease specific core sets for mental conditions were included [2]. The rationale for this is that although mental conditions are predictors of long-term sickness absence [17], these conditions are underrepresented in current practice-based instruments. Finally, the list was expanded with one item derived from a non-ICF practice-based instrument, the Dutch Checklist Experienced Limitations [18] which could easily be converted to an ICF d-code. The preliminary list consisted of 184 categories.

The research team excluded 34 categories prior to the first round because these categories were clearly not relevant within a work context according to their experts opinion, such as toileting and dressing. They divided the resulting 150 unique second level categories in six sections (partly) based on Dutch practice-based non-ICF instruments: 1) personal functioning, 2) social functioning, 3) dynamic movements, 4) static postures, 5) external (work) factors and 6) functions (Supplementary materials Appendix 1). We applied these sections because our experts are used to structure their assessment of the work capacity around these domains. Sections 1–4 included codes related to the ICF component ‘activity and participation’ (i.e. d-codes), Sect. 5 included codes related to ICF component ‘environmental factors’ (i.e. e-codes), and Sect. 6 included factors related to ICF component ‘body functions’ (i.e. b-codes).

While composing the list of ICF categories, the research team judged, based on their experts’ opinion that the e-codes from the ICF were insufficient for the assessment of work capacity and return to work possibilities. That is, they primarily relate to the physical environment, while ignoring the psychosocial environment. Also, although they could be applied to the working environment, they do not specifically refer to work. As such, the composed ICF list was supplemented with a list of 27 work-related environmental factors with underlying factors on a more detailed level (Supplementary materials Appendix 2) [19]. Together, they formed the input for the Delphi study.

### Delphi protocol

The total modified Delphi study started with two large e-mail rounds based on Delphi techniques, i.e. round 1 and 2, two virtual meetings, i.e. round 3 and 4, and an intermediate small e-mail round between round 3 and 4. Round 1 until 3 were planned before the start of the Delphi study. After round 3, it became clear that there were concerns regarding the first draft of the instrument among some of the experts. At that point it was decided to organize an additional small e-mail round and a virtual round. For further information see ‘Results – Draft of the instrument’.

Before the start of the Delphi rounds the research team determined several consensus rules. The literature provides no clear indications on consensus rules, thus the research team determined the consensus rules based on what they thought to be appropriate. Consensus regarding relevance was reached when at least 80% of the experts considered the item to be relevant. If this threshold was reached, consensus regarding interpretability was calculated. When at least 70% of the experts considered the ICF category to be interpretable this resulted in preliminary inclusion in the instrument. When less than 70% of the experts considered the item to be interpretable, this specific item was presented again in the next Delphi round. When 70%-79% of the experts considered the item to be relevant, this specific item was presented again in the next Delphi round. When less than 70% considered the item to be relevant, this resulted in preliminary exclusion. In the virtual meetings the same consensus rules as in the previous e-mail rounds were applied. In case the expert team could not reach consensus after the four rounds the research team would take the decisive decision.

During *Delphi round 1* experts were asked to indicate in an online questionnaire relevance for the assessment of the work capacity as well as interpretability of the 150 items (i.e. 3^rd^ level ICF categories including the underlying 4^th^ level categories) of the input list. Relevance could be indicated on a five-point Likert scale ranging from ‘not important at all’ to ‘extremely important’ and interpretability on a five-point Likert scale ranging from ‘not at all’ to ‘to a very high degree’. Experts got the opportunity to explain their choices in an open text field. Experts were also asked for their preference on the level of detail of the work-related environmental factors (i.e. on the level of subdivision, item or sub-item) (Supplementary materials Appendix 2). After Delphi round 1 the research team processed the data in three steps. First, relevance and interpretability were dichotomized in ‘not relevant’ and ‘relevant’, and ‘not interpretable’ and ‘interpretable’, respectively. Second, consensus for every item was calculated. Third, following the consensus rules, decisions were made on preliminary in- or exclusion, as well as the need to present the specific item in the next round. The preferred level of detail of the work-related environmental factors of the experts was assessed and would be the level of detail of presenting the work-related environmental factors in the next round.

During *Delphi round 2* experts were asked to indicate in an online questionnaire relevance for those items on which no consensus was reached in Delphi round 1, either in terms of relevance, or in terms of interpretability. This time the items were accompanied by consensus information (quantitative) and a narrative summary of the argumentation of the experts (qualitative) based on Delphi round 1. In addition, the experts were requested to indicate the relevance of a total of 46 items, i.e. e-codes, for the assessment of return to work possibilities. Finally, in this Delphi round the 27 work-related environmental factors were presented to the experts including underlying factors on a more detailed level. They were asked to indicate relevance of these factors. The research team processed the data of round 2 in the same way as those of round 1. Moreover, the research team took a critical look at the preliminary instrument on its relevance for practice.

Thereafter, a virtual round was arranged, *Delphi round 3*, aiming at reflecting on the preliminary list of items after two Delphi rounds. The virtual round was moderated by one of the members of the research team, who has ample experience in leading group discussions, also within the context of Delphi studies. An inventory was held on whether there are ICF categories missing and whether there are preliminary included ICF categories that deemed unnecessary. An expert who missed an ICF category had the opportunity to explain why he/she thought this category was important and a vote was held. The consensus rules were the same as those applied in round 1 and 2. After this virtual round the research team composed a first draft of the instrument, which was shared with the expert team. An additional e-mail round and a virtual round were needed as some of the experts raised their concerns regarding the first draft of the instrument (see ‘Results – Draft of the instrument’). Two working groups involving representatives of the three professional groups prepared a next virtual round, *Delphi round 4*.

## Results

The 12 experts filled in the online questionnaires belonging to Delphi round 1, 2 and 3. During the additional e-mail round 11 experts participated and 10 experts attended Delphi round 4. Halfway in the Delphi study one of the experts has been replaced by another expert from the same professional group, because of retirement.

### Delphi round 1

**Fig. 1:**
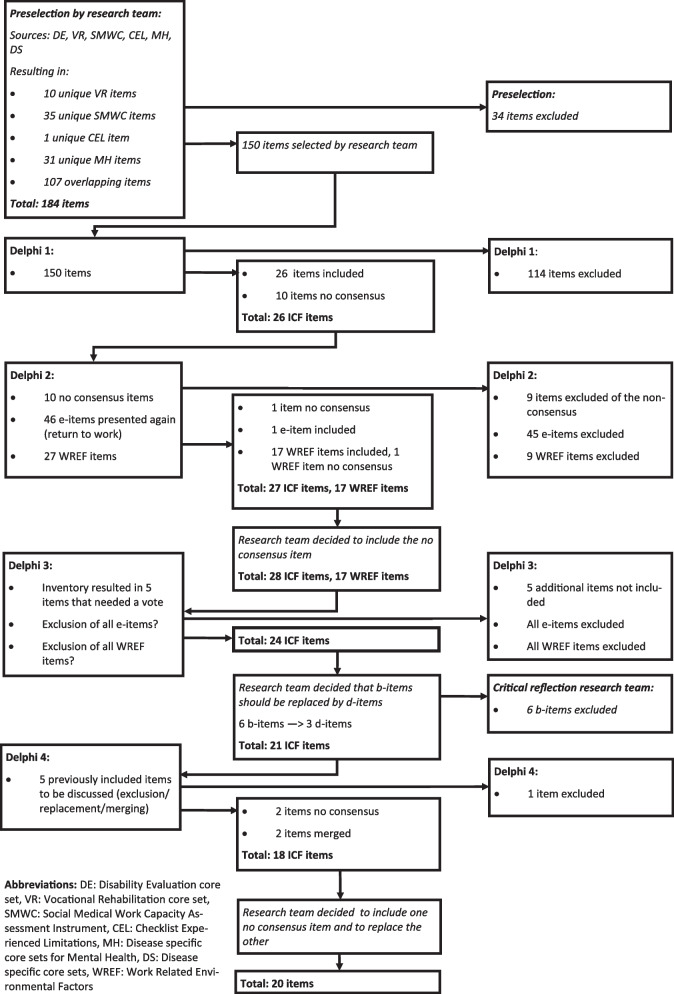
Flow of the item list

In Delphi round 1 the experts reached consensus on preliminary inclusion and exclusion of 26 items and 114 items, respectively (Fig. 1. Flow of the item list). It was not necessary to present these items in a subsequent round. The experts did not reach consensus on ten items and consequently these items were presented again in Delphi round 2. For nine of these items there was no consensus on the relevance and for two of these items there was no consensus on interpretability.

### Delphi round 2

In Delphi round 2 the experts reached consensus on preliminary exclusion of nine of the ten items that were presented for the second time. The experts did not reach consensus on the final item. In Delphi round 2, 46 items, i.e. e-codes, were presented again to the experts with the question to indicate relevance for the assessment of return to work possibilities. This resulted in one additional preliminary inclusion. Presentation of the work-related environmental factors during this Delphi round resulted in 17 preliminary inclusions, nine preliminary exclusions and one item on which no consensus was reached among the experts. The original list of 150 unique ICF 3-digit codes was reduced to a list of 27 items and one undecided item after Delphi round 1 and 2. This last item remained in the list.

### Reflection of the research team

It was noticed by the research team that 1) the list comprised overlapping items (i.e. d160 Focusing attention and b140 Attention functions), 2) one item had been excluded by the expert team, which was considered to be relevant based on their expert opinion (i.e. b455 Exercise tolerance functions), and 3) the list of included e-codes regarding external (work) factors seemed inadequate to provide a full overview of relevant workplace factors, and that it would be more helpful in an additional document that could accompany the instrument. The research team also reflected on the remaining 17 items from the additional list of work-related environmental factors. They concluded that it would be more appropriate if the complete list of work-related factors would be retained, not in the instrument itself, but in an additional document. They decided to pay particular attention to these issues in *Delphi round 3*.

### Delphi round 3

The inventory at the beginning of *Delphi round 3* resulted in three ICF codes that had already been presented in the previous Delphi rounds (e.g. d170 Writing) that were put to vote again. The votes resulted in exclusion because none of the items reached the consensus threshold for inclusion of 80%. Furthermore, a vote was held on the overlapping items and the missing item identified by the research team (see ‘Reflection of the research team’). With regard to the first set of overlapping items the experts’ opinions were divided on which one to include (50%-50%). As the experts already reached consensus on inclusion of these items in the previous Delphi rounds, it was decided to retain both items. With regard to the other set of overlapping items the experts reached consensus (83% preferred to keep both items) that both items should be retained. The experts disagreed on inclusion of the missing item identified by the research team (50%-50%) and therefore it was decided not to include this item. The experts furthermore reached a consensus that the e-codes (83% agreed) and the additional list of work-related environmental factors (92% agreed) should not be part of the instrument.

### Draft of the instrument

Based on these results a draft of the instrument was developed, which was shared with the expert team. After legal consultation and discussion with the expert team it was decided to replace the remaining b-codes, i.e. codes concerning body functions, by equivalent d-codes, i.e. codes concerning activities and participation, as the instrument would in practice also be interpreted by LEs, who lack a medical background, and employers. As such no medical information should be revealed within the instrument. Further, the expert team expressed their concerns regarding items that were either missing or were considered unpractical in daily occupational health care unnecessary. It became apparent that an additional virtual round was needed to address these concerns.

### Delphi round 4

In this virtual round the experts were invited to give their opinion on five previously included items regarding in- or exclusion, replacement or merging. The experts reached consensus on one item to be excluded and on merging of two other items. No consensus was reached on the final two items. Afterwards the research team decided to retain one of these items and to replace the other. This decision was based on the experts’ arguments and opinions during this fourth round.

### Final instrument

The final instrument (Table 1 and Supplementary materials Table S1) consists of 20 items, of which six items for personal functioning, seven items for social functioning and seven items for physical functioning.

**Table 1:** List of ICF-items in the final instrument

Personal functioning	
1	d159 Basic learning, other specified and unspecified
2	d160 Focusing attention
3	d175 Solving problems
4	d177 Making decisions
5	d220 Undertaking multiple tasks
6	d240 Handling stress and other psychological demands
Social functioning	
7	d110 Watching
8	d115 Listening
9	d120 Other purposeful sensing
10	d330 Speaking
11	d470 Using transportation + d475 Driving
12	d720 Complex interpersonal interactions
13	d740 Formal relationships
Physical functioning	
14	d410 Changing basic body position
15	d430 Lifting and carrying objects
16	d440 Fine hand use
17	d445 Hand and arm use
18	d450 Walking
19	d451 Going up and down stairs
20	d415 Maintaining a body position

## Discussion

The current study aimed to reach multidisciplinary consensus among professionals in the domain of occupational healthcare on the content of an ICF-based instrument for the assessment of the work capacity and return to work possibilities of employees on sick leave by conducting a Delphi study. The four Delphi rounds resulted in 20 items to be included in the instrument.

### Comparison with other ICF-based instruments and core sets within the field of occupational health

The developed instrument in this modified Delphi study is based on several ICF core sets and practice instruments for the specific purpose of the assessment of work capacity and guidance in return to work. In line with the ICF framework items are classified within domains of personal, social and physical functioning. Nevertheless, items within these domains closely relate to the domains physical, mental and emotional, as specified in the definition of work capacity presented in the introduction (i.e. ‘the overall ability of an individual to perform the physical, mental and emotional tasks that are needed for the requirements of a particular job, or class of jobs’) [3]. This indicates a generally adequate face validity of the content of the instrument for the assessment of work capacity. Only the emotional domain is limited, because it is covered by just one item, i.e. d240 Handling stress and other psychological demands. The planned instrument, however, allows for the possibility to include ICF codes beyond the ones that are included.

Also, the instrument is comparable to the disability evaluation core set [15], the vocational rehabilitation core set [16] and the Social Medical Work Capacity assessment instrument [2] as they all include ICF items that relate to personal, social and physical functioning (domains based on Dutch practice-based instruments for the assessment of work capacity). The included items for personal and social functioning are also included in the disease specific core sets for mental health. All in all, the current instrument is much briefer than the vocational rehabilitation core set (*n* = 90) [16] and the Social Medical Work Capacity assessment instrument (*n* = 129) [2]. It is comparable in size with the disability evaluation core set (*n* = 20) [15], which was specifically developed for medical advisors in social security to facilitate them to decide whether a person should be granted disability benefits, which is a different application than that of the current instrument.

### Application of the instrument and its strengths and weaknesses

The current study resulted in 20 items to be included in an ICF-based instrument for the assessment of the work capacity and return to work possibilities of employees on sick leave. The instrument is meant for the assessment of the work capacity of an employee on sick leave for the benefit of optimal guidance in return to work. By using ICF as shared conceptual framework and common language the instrument should contribute to better communication between OPs, LEs, and IPs and improve their collaboration.

The instrument is developed for the Dutch occupational healthcare system. It should be noted that this system has some specific characteristics, which are not necessarily applicable to the systems in other countries. One specific element is the involvement of labour experts in the return to work process of employees on sick leave. Labour experts have an analytical or human-scientific educational background followed by professional training for the specific labour expert profession [20]. According to the Dutch privacy law, they are not allowed to have access to medical information. For the development of the instrument this meant that ICF b-codes concerning body functions had to be removed from the instrument because they entail medical information.

The developed instrument is expected to facilitate interdisciplinary and intradisciplinary communication and collaboration because of the use of the ICF as a shared conceptual framework between OPs, LEs, and IPs. Adopting the ICF as a common language between occupational healthcare professionals also offers opportunities for better communication with medical specialists in curative care, as ICF core sets are already applied in clinical practice, and, as such, the ICF could play a role in further development of clinical work-integrating care. The developed instrument is furthermore limited in length, which makes it useful for daily practice.

In the current Delphi study we explored a way to include items on work-related external factors. ICF e-codes that may pertain to the work situation of an employee and a list of work-related environmental factors compiled by Heerkens et al. [19] were presented to the experts. The latter has been established through a bottom-up approach, including feedback from researchers, and was based on the literature. It includes the subdivisions task content, terms of employment, social relationships at work and working conditions. Both the ICF e-codes and the list of non-ICF work-related environmental factors did not lead to satisfying results according to the experts because the remaining items did not cover all relevant aspects of an employees’ work situation that may impact the return to work process, which may have to do with the wording of the ICF e-codes and the non-ICF work-related environmental factors. The notion that some factors that could be relevant within the context of occupational healthcare are not included in the ICF has been acknowledged in previous research (e.g. [21, 22]). Even more, in the context of technological developments and increasingly flexible labour markets, other work factors may become relevant such as being available for work continuously and increasingly insecure and flexible employment contracts. Within a context with labour market policies aiming at individuals experiencing health problems to remain active in work [3], it is of utmost importance to have attention for factors in the work environment that facilitate or hinder re-integration of employees on sick leave. It is worth to further investigate possibilities for integration of work-related environmental factors in a new version of the instrument, to shape return to work guidance from a biopsychosocial perspective.

Another weakness of the instrument may be that personal factors are not included, whereas it is known from previous research that they are important in the return to work process. To illustrate, a recent study of De Wit and colleagues showed that both occupational physicians and insurance physicians considered personal factors, such as expectations regarding recovery or return to work, self-efficacy and coping strategies, to have an important influence on work participation of employees with health problems [23]. However, personal factors are also not included in the disability evaluation core set, and in a validation study medical examiners did not consider them to be missing [24]. As the developed instrument would be read and interpreted by both non-medical professionals (i.e. labour experts) and by employers, due to privacy issues, it was concluded that personal factors could not be part of the current instrument.

### Methodological considerations

A strength of this Delphi study is the multidisciplinary composition of the expert panel, with all relevant professional groups involved in the assessment of work capacity and return to work possibilities of employees on sick leave being represented. Another strength is that we enriched the traditional Delphi technique characterized by collecting opinions anonymously (formal Delphi rounds 1 and 2) with structured face to face meetings (i.e. applied in an online setting) that allowed for more direct exchange between the experts (Delphi round 3 and 4). In first instance the formal Delphi rounds allowed the experts to give their opinion without social pressure that could play a role in face to face meetings. The virtual rounds in second instance allowed the experts to interact with each other and enabled them to put themselves in someone else’s position. Although during these occasions social pressure and its potentially negative consequences cannot be ruled out, the moderator was an experienced process leader (last author), who safeguarded the structure and let everyone speak. Due to the COVID-19 circumstances and social distancing measures that were in place at the time of data collection we were restricted to online meetings. This, however, revealed not to be problematic, which can be derived from the fruitful discussions that have taken place during these sessions. In general, traditional Delphi principles were followed in our modified Delphi study. Although there are no clear guidelines in the literature on the number of experts to include in a Delphi study, and there is much variation in the numbers applied within previous Delphi studies, it should be noted that with twelve experts we had a relatively small expert team [25]. A larger number of experts could have contributed to a more reliable overall judgment on the relevance and interpretability of the items. Instead the research team had a larger role than usual at some points. To illustrate, the research team, critically reflected on the item list after two Delphi rounds and used their ideas as input for the third Delphi round. As the research team consisted of experts within occupational healthcare themselves, we think this is justified as well as of added value. All in all, this resulted in a Delphi study that seemed to fit the Dutch occupational healthcare practice.

### Implications for research and practice

The content of the instrument for the assessment of work capacity and return to work possibilities was developed with involvement of representatives of all relevant professions in the domain of occupational healthcare. The instrument should now further be developed, validated and evaluated. In the further development of the instrument it is important to take into account the perspectives of employees and employers, being important stakeholders when it comes to implementation of the instrument in practice. The instrument should be clear and transparent in reporting the employee’s work capacity and return to work possibilities. In addition, the instrument should be further developed into a user-friendly (digital) instrument that facilitates return to work and is uniformly interpretable across different professions in occupational health. Extensive evaluation and testing by occupational health professionals is necessary. Also training of all professionals involved in the use of the instrument for the assessment of work capacity and return to work possibilities becomes important for successful implementation of the instrument in occupational healthcare practice.

## Conclusions

The current study consisting of four Delphi rounds resulted in 20 items that are considered minimally needed in an instrument for the assessment of the work capacity and return to work possibilities of employees on sick leave. This set of items forms the core of an ICF-based instrument, which is currently under development with stakeholders in the Dutch field of occupational healthcare to prepare for its final implementation in practice.

## Supplementary Information

**Additional file 1.**

## Data Availability

The datasets used and/or analysed during the current study are available from the corresponding author on reasonable request.

## Abbreviations

ICF: International Classification of Functioning, Disability and Health
IP: Insurance physician
LE: Labour expert
OP: Occupational physician
