# Supplementary material for: Development of the core of an ICF-based instrument for the assessment of work capacity and guidance in return to work of employees on sick leave: a multidisciplinary modified Delphi study
Source: BMC Public Health. 2022 Dec 28;22:2449. doi: 10.1186/s12889-022-14653-0 (PMC9795420; doi:10.1186/s12889-022-14653-0)
Supplement: Supplementary file 1 — Additional file 1. [file 12889_2022_14653_MOESM1_ESM.docx]

### **Supplementary materials belonging to ‘Development of the core of an ICF-based instrument for the assessment of work capacity and guidance in return to work of employees on sick leave: a multidisciplinary modified Delphi study’**

**Appendix 1. Overview of ICF-items presented in Delphi round 1**

**Appendix 2. Overview of work-related environmental factors**

**Table S1. List of ICF-items in the final instrument classified according to previous ICF core sets and practice-based instruments**

**Appendix 1. Overview of ICF-items presented in Delphi round 1**

**Personal functioning**

1. d155 Acquiring skills
2. d160 Focusing attention
3. d163 Thinking
4. d175 Solving problems
5. d177 Making decisions
6. d210 Undertaking a single task
7. d220 Undertaking multiple tasks
8. d230 Carrying out daily routine
9. d240 Handling stress and other psychological demands
10. d310 Communicating with - receiving - spoken messages
11. d325 Communicating with - receiving - written messages
12. d345 Writing messages

**Social functioning**

1. d110 Watching
2. d115 Listening
3. d120 Other purposeful sensing
4. d166 Reading
5. d170 Writing
6. d172 Calculating
7. d250 Managing one’s own behaviour
8. d315 Communicating with - receiving - nonverbal messages
9. d330 Speaking
10. d335 Producing nonverbal messages
11. d340 Producing messages in formal sign language
12. d350 Conversation
13. d355 Discussion
14. d360 Using communication devices and techniques
15. d470 Using transportation
16. d475 Driving
17. d710 Basic interpersonal interactions
18. d720 Complex interpersonal interactions
19. d730 Relating with strangers
20. d740 Formal relationships

**Dynamic movements**

1. d410 Changing basic body position
2. d420 Transferring oneself
3. d430 Lifting and carrying objects
4. d440 Fine hand use
5. d445 Hand and arm use
6. d446 Fine foot use
7. d450 Walking
8. d451 Going up and down stairs
9. d455 Moving around
10. d465 Moving around using equipment

**Static postures**

1. d415 Maintaining a body position

**External (work) factors**

1. e110 Products or substances for personal consumption
2. e115 Products and technology for personal use in daily living
3. e120 Products and technology for personal indoor and outdoor mobility and transportation
4. e125 Products and technology for communication
5. e130 Products and technology for education
6. e135 Products and technology for employment
7. e150 Design, construction and building products and technology of buildings for public use
8. e155 Design, construction and building products and technology of buildings for private use
9. e165 Assets
10. e225 Climate
11. e240 Light
12. e245 Time-related changes
13. e250 Sound
14. e255 Vibration
15. e260 Air quality
16. e310 Immediate family
17. e315 Extended family
18. e320 Friends
19. e325 Acquaintances, peers, colleagues, neighbours and community members
20. e330 People in positions of authority
21. e340 Personal care providers and personal assistants
22. e355 Health professionals
23. e360 Other professionals
24. e410 Individual attitudes of immediate family members
25. e415 Individual attitudes of extended family members
26. e420 Individual attitudes of friends
27. e425 Individual attitudes of acquaintances, peers, colleagues, neighbours and community members
28. e430 Individual attitudes of people in positions of authority
29. e440 Individual attitudes of personal care providers and personal assistants
30. e450 Individual attitudes of health professionals
31. e455 Individual attitudes of other professionals
32. e460 Societal attitudes
33. e465 Social norms, practices and ideologies
34. e525 Housing services, systems and policies
35. e535 Communication services, systems and policies
36. e540 Transportation services, systems and policies
37. e545 Civil protection services, systems and policies
38. e550 Legal services, systems and policies
39. e555 Associations and organizational services, systems and policies
40. e560 Media services, systems and policies
41. e565 Economic services, systems and policies
42. e570 Social security services, systems and policies
43. e575 General social support services, systems and policies
44. e580 Health services, systems and policies
45. e585 Education and training services, systems and policies
46. e590 Labour and employment services, systems and policies

**Functions**

1. b110 Consciousness functions
2. b114 Orientation functions
3. b117 Intellectual functions
4. b122 Global psychosocial functions
5. b125 Dispositions and intra-personal functions
6. b126 Temperament and personality functions
7. b130 Energy and drive functions
8. b134 Sleep functions
9. b140 Attention functions
10. b144 Memory functions
11. b147 Psychomotor functions
12. b152 Emotional functions
13. b156 Perceptual functions
14. b160 Thought functions
15. b163 Basic cognitive functions
16. b164 Higher-level cognitive functions
17. b167 Mental functions of language
18. b172 Calculation functions
19. b176 Mental function of sequencing complex movements
20. b180 Experience of self and time functions
21. b210 Seeing functions
22. b230 Hearing functions
23. b235 Vestibular functions
24. b240 Sensations associated with hearing and vestibular function
25. b260 Proprioceptive function
26. b265 Touch function
27. b270 Sensory functions related to temperature and other stimuli
28. b280 Sensation of pain
29. b310 Voice functions
30. b320 Articulation functions
31. b330 Fluency and rhythm of speech functions
32. b410 Heart functions
33. b415 Blood vessel functions
34. b420 Blood pressure functions
35. b430 Haematological system functions
36. b435 Immunological system functions
37. b440 Respiration functions
38. b445 Respiratory muscle functions
39. b450 Additional functions of the respiratory system
40. b455 Exercise tolerance functions
41. b460 Sensations associated with cardiovascular and respiratory functions
42. b525 Defecation functions
43. b530 Weight maintenance functions
44. b535 Sensations associated with the digestive system
45. b540 General metabolic functions
46. b550 Thermoregulatory functions
47. b555 Endocrine gland functions
48. b620 Urination functions
49. b640 Sexual functions
50. b710 Mobility of joint functions
51. b715 Stability of joint functions
52. b730 Muscle power functions
53. b735 Muscle tone functions
54. b740 Muscle endurance functions
55. b750 Motor reflex functions
56. b755 Involuntary movement reaction functions
57. b760 Control of voluntary movement functions
58. b765 Involuntary movement functions
59. b780 Sensations related to muscles and movement functions
60. b810 Protective functions of the skin
61. b820 Repair functions of the skin

**Appendix 2. Overview of work-related environmental factors**

**Task content**

1. Autonomy / job control
2. Decision authority
3. Job demands
   - Emotional demands
   - Mental demands
   - Physical demands
4. Job rotation
5. Job tasks
6. Responsibilities
7. Role ambiguity
8. Skills required for task
9. Variation
10. Work pace

**Terms of employment**

1. Career opportunities / possibilities
   - Possibilities for education / development
   - Promotion possibilities / opportunities
2. Contract / remuneration package
   - Job security / certainty
   - Salary / remuneration package
3. Secondary benefits
   - Child care provided by organisation
   - Leave arrangements
4. Working time arrangements
   - (Flexibility in) working hours
   - (Flexibility in) working schedules
   - Shift work

**Social relationships at work**

1. Attitude
   - Of colleague
   - Of subordinate
   - Of superior
2. Communication
   - Communication skills of employer / superior
   - Means of communication
   - Structure in communication / consultative structure (participants, frequency / duration)
3. Conflict
   - With superior
   - With colleague
   - With subordinate
4. Management style
   - General within organisation
   - Of superior
5. Support
   - From colleague
   - From subordinate
   - From superior

**Working conditions**

1. Biological and chemical agents
2. Dangers / safety
3. Emotional conditions workplace
   - Discrimination
   - Sexual harassment
   - Violence
4. Ergonomic conditions workplace
   - Accessibility of building
   - Furniture
   - Input devices for computers: mouse-like devices, computer joysticks, keyboard
   - Tools and machinery
5. Hygiene
6. Mental conditions workplace
   - Boring or defiant work
   - Time pressure
7. Personal safety equipment
8. Physical conditions workplace
   - Dust
   - Humidity
   - Light
   - Noice
   - Radiation
   - Temperature
   - Vibrations

**Table S1. List of ICF-items in the final instrument classified according to previous ICF core sets and practice-based instruments**

| **ICF-items in the final instrument** | | **DE** | **VR** | **SMWC** | **CEL** | **DS** | **MH** |
| --- | --- | --- | --- | --- | --- | --- | --- |
| **Personal functioning** | | | | | | | |
| 1 | d159 Basic learning, other specified and unspecified |  |  | X |  |  |  |
| 2 | d160 Focusing attention |  | X | X | X |  | X |
| 3 | d175 Solving problems |  | X |  |  |  | X |
| 4 | d177 Making decisions | X | X |  | X |  | X |
| 5 | d220 Undertaking multiple tasks | X | X | X* | X* | X* | X |
| 6 | d240 Handling stress and other psychological demands | X | X | X | X |  | X |
| **Social functioning** | | | | | | | |
| 7 | d110 Watching | X |  | X | X |  | X |
| 8 | d115 Listening | X |  | X | X |  | X |
| 9 | d120 Other purposeful sensing |  |  | X* |  |  |  |
| 10 | d330 Speaking |  |  | X |  |  | X |
| 11 | d470 Using transportation + d475 Driving | X (only d470) | X | X |  |  | X |
| 12 | d720 Complex interpersonal interactions | X | X | X | X |  | X |
| 13 | d740 Formal relationships |  | X |  |  |  | X |
| **Physical functioning** | | | | | | | |
| 14 | d410 Changing basic body position | X | X | X* | X* |  |  |
| 15 | d430 Lifting and carrying objects | X | X | X | X* |  |  |
| 16 | d440 Fine hand use | X | X | X | X |  |  |
| 17 | d445 Hand and arm use | X | X | X | X* |  |  |
| 18 | d450 Walking | X | X | X | X |  |  |
| 19 | d451 Going up and down stairs |  |  |  | X |  |  |
| 20 | d415 Maintaining a body position | X | X | X | X* |  |  |

* Only included as ICF fourth level category

DE = Disability Evaluation core set; VR = Vocational Rehabilitation core set; SMWC = Social Medical Work Capacity assessment instrument; CEL = Checklist Experienced Limitations; DS = Disease Specific core sets; MH = disease specific core sets for Mental Health
